# Computed Tomography Angiographic Morphometry of the Maxillary Artery in Relation to Mandibular Landmarks

**DOI:** 10.3390/diagnostics16152406

**Published:** 2026-07-30

**Authors:** Viviana Dincă, Mugurel Constantin Rusu, Răzvan Costin Tudose, Adela Gabriela Stănescu, Adelina Maria Jianu, George Triantafyllou, Maria Piagkou, Iulian Brezean

**Affiliations:** 1 Division of Anatomy, Department 1, Faculty of Dentistry, “Carol Davila” University of Medicine and Pharmacy, 020021 Bucharest, Romania; viviana.dinca@drd.umfcd.ro (V.D.); razvan-costin.tudose0721@stud.umfcd.ro (R.C.T.); adela-gabriela.stanescu2024@stud.umfcd.ro (A.G.S.); 2 Department of Anatomy and Embryology, Victor Babeș University of Medicine and Pharmacy, 300041 Timișoara, Romania; adelina.jianu@umft.ro; 3 Department of Radiology and Medical Imaging, Emergency Clinical Municipal Hospital, 300254 Timișoara, Romania; 4 Department of Anatomy, School of Medicine, Faculty of Health Sciences, National and Kapodistrian University of Athens, 75 Mikras Asias str, Goudi, 11527 Athens, Greecemapian@med.uoa.gr (M.P.); 5 Department of General Surgery, “Carol Davila” University of Medicine and Pharmacy, Cantacuzino Clinical Hospital, 030167 Bucharest, Romania; iulian.brezean@umfcd.ro

**Keywords:** maxillary artery, mandibular lingula, gonial angle, mandibular notch, lateral pterygoid muscle, computed tomography angiography, infratemporal fossa, looping

## Abstract

**Background/Objectives**: To establish normative CTA (computed tomography angiography) morphometry of the maxillary artery (MA) relative to the lateral pterygoid muscle (LPM) and mandibular landmarks in a European population. **Methods:** Bilateral CTA datasets from 164 patients (328 sides; mean age 67.2 ± 13.0 years) were analysed retrospectively. The MA course was classified relative to the inferior head of the LPM as superficial (S) or deep (D); patients were assigned to three bilateral types (Type 1: S + S; Type 2: D + D; Type 3: S + D), and MA–gonial angle, MA–mandibular notch, and MA–lingula distances were measured. **Results:** The MA was superficial in 61.0% of sides, with no transpterygoid course; Type 1 predominated (48.8%). The MA–gonial angle distance was 34.53 ± 5.31 mm, with marked sex dimorphism (males: 36.74 mm vs. females: 31.35 mm; *p* < 0.0001). Looping occurred on at least one side in 34.8% of patients, and the MA lay superior to the mandibular notch in 83.4% of superficial sides. The MA–lingula distance was 8.03 ± 2.54 mm; non-retrotuberosity looping was strongly associated with proximity below 5 mm (OR = 9.95). **Conclusions:** A three-type bilateral classification of the MA course, the first normative MA–gonial angle dataset, and the looping–proximity association provide actionable data for surgical risk stratification in infratemporal and mandibular ramus procedures.

## 1. Introduction

The maxillary artery (MA) is the larger terminal branch of the external carotid artery (ECA), supplying the deep face through approximately 14 named collateral branches [1]. Arising at the mandibular neck, it traverses the infratemporal fossa (ITF) and terminates in the pterygopalatine fossa (PPF) as the sphenopalatine artery. Three segments are classically recognised based on the artery’s relationship with the LPM [2,3].

The most extensively characterised topographic feature of the MA is its course relative to the inferior head of the LPM: superficial (lateral), deep (medial), or intramuscular. Two recent meta-analyses pooling data from over 5900 arteries document marked ethnic variation: approximately 90.9% superficial in Asian samples, 58.1–62.9% in White/European samples, and 56.7–63.1% in African samples [3,4,5]. Bilateral asymmetry occurs in approximately 21–26% of individuals [6,7], yet published studies invariably report the MA–LPM relationship as a per-side variable, without characterising patients according to their bilateral topographic pattern.

Several anatomical relationships of direct surgical relevance remain poorly characterised at the population level. The proximity of the MA to mandibular landmarks has been investigated in both cadaveric studies and a single 3D CT study. Hwang et al. (2014) reconstructed the MA, LPM, and mandibular ramus in 100 Korean patients and quantified relationships with the coronoid process, condyle, and mandibular notch [8]. However, they did not evaluate MA looping, the MA–gonial angle distance, or the MA–lingula distance. Balcioglu et al. (2010) and Warui et al. (2023) provided cadaveric data relevant to ramus and notch anatomy but no systematic CTA-based MA–lingula dataset [9,10]. More importantly, MA looping in the ITF, a finding with direct implications for the safety of inferior alveolar nerve (IAN) blocks and subcondylar surgery, has been reported exclusively in isolated CTA case reports [11,12,13], with no population-level prevalence data available. Similarly, the MA–gonial angle distance has not been systematically measured in any patient population. However, the gonial angle was incidentally referenced as a distance marker in a single CTA case report on maxillofacial trunk variants [14].

The present study addresses these gaps through a retrospective bilateral CTA morphometric analysis of 164 patients. We (1) introduce a three-type bilateral classification of the MA–LPM relationship; (2) provide the first normative dataset for the MA–gonial angle; (3) establish the population-level prevalence and topographic classification of MA looping in the ITF; (4) present the first systematic data on the MA–lingula distance; and (5) demonstrate a clinically critical association between non-retrotuberosity looping and close MA–lingula proximity. These landmarks carry direct procedural relevance: the mandibular lingula and gonial angle for the inferior alveolar nerve (IAN) block and sagittal split ramus osteotomy, the mandibular notch for subcondylar and intraoral vertical ramus osteotomies, and the retrotuberosity segment for pterygomaxillary disjunction, Le Fort I osteotomy, and image-guided approaches to the pterygopalatine fossa, including maxillary nerve block.

## 2. Material and Methods

### 2.1. Study Design and Ethical Considerations

This retrospective cross-sectional study of archived CTA data was conducted in accordance with the Declaration of Helsinki and approved by the Institutional Review Board of the Emergency Clinical Municipal Hospital of Timișoara (approval number E-4247/15 October 2025). Informed consent was obtained from all subjects involved in the study. This consent covered the retrospective use of anonymized imaging data for research and publication, in accordance with Regulation (EU) 2016/679 (GDPR).

### 2.2. Patient Selection

Archived CTA studies were screened. Inclusion criteria: (1) age ≥ 18 years; (2) technically adequate CTA with opacification of the ECA and its branches; and (3) complete bilateral MA visualisation from the ECA origin to the PPF. Exclusion criteria: (1) prior facial or mandibular surgery; (2) known vascular anomaly pre-study; (3) significant artefact precluding measurement; and (4) non-diagnostic MA opacification. After screening, 164 patients (328 sides) were included. No a priori sample-size or power calculation was performed; the study enrolled all consecutive eligible datasets available during the study period.

### 2.3. Imaging Protocol and Reconstruction

All studies were performed on a 32-slice MDCT scanner (Aquilion 32; Toshiba Medical Systems Corporation, Otawara, Japan) with intravenous iodinated contrast (100 mL; injection rate 4 mL/s; bolus-tracking trigger 150 HU at the aortic arch level). Images were reconstructed at 1 mm slice thickness with a 0.5 mm increment. Multiplanar reconstruction (MPR) and three-dimensional volume-rendered (3D-VR) images were generated using Horos v4.0. All measurements were taken using the built-in digital calliper on axial, coronal, and oblique MPR images, with verification on 3D-VR reconstructions.

### 2.4. Variables Assessed

**Figure 1:**
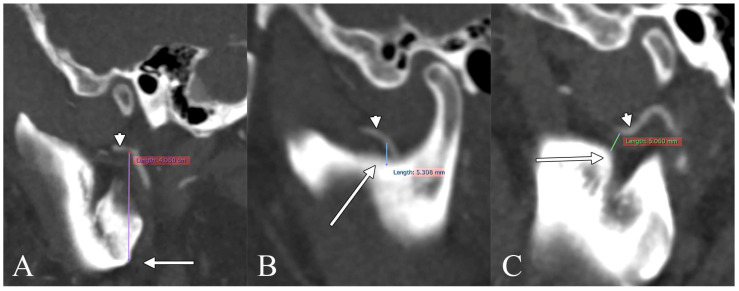
Measurements of distances of the maxillary artery (MA, arrowheads) to specific landmarks on sagittal CTA slices through the mandibular ramus, left side, lateral views (single case). (**A**) MA–gonial angle (arrow) distance. (**B**) MA–mandibular notch (arrow) distance. (**C**) MA–lingula (arrow) distance.

Five variables were assessed bilaterally on each patient:

Variable 1—MA–gonial angle distance (MA–GA; mm): The shortest distance from the MA trunk to the most postero-inferior point of the mandibular ramus-body junction (gonial angle), measured on axial/oblique-coronal and sagittal MPR (Figure 1A). Measurable regardless of the MA course. The gonial angle has not previously been used as a systematic measurement variable for MA localisation. However, it was cited as a single descriptive distance in a CTA case report [14].

Variable 2—Directional MA–mandibular notch position (MA–MN; mm): Perpendicular signed distance from the MA to the mandibular notch plane on coronal MPR. Positive values indicate that the MA is superior to the notch plane; negative values indicate inferior placement. Measured on superficial-course sides only (Figure 1B), as the clinical hazard is relevant only when the MA passes lateral to the LPM [8,9].

Variable 3—MA looping in the ITF: Binary (absent/present), with morphological characterisation and topographic classification into two groups: (a) interpterygoid triangle loops, within the pterygoid compartment (medial, inferior, U, reversed U, coil loops); and (b) retrotuberosity loops, posterior to the maxillary tuberosity. Combined configurations were recorded. Retrotuberosity sides were excluded from all loop–lingula analyses on anatomical grounds, as these loops cannot approach the lingula.

Variable 4—MA course relative to the LPM: Classified as superficial/lateral (S), deep/medial (D), or through/intramuscular (T) on axial MPR [4,5]. Bilateral combinations were coded as Type 1 (S + S), Type 2 (D + D), or Type 3 (S + D).

Variable 5—Minimum MA–lingula distance (MA–Ling; mm): Shortest distance from the MA to the mandibular lingula (spine of Spix) on coronal and sagittal MPR (Figure 1C), restricted to non-retrotuberosity superficial sides. Thresholds of <5 mm and <3 mm were used to define close and very close proximity, respectively.

### 2.5. Interobserver Agreement

A random 20% subsample (n = 33) was independently re-measured by a blinded senior radiologist (5 years of experience in vascular head and neck imaging). Intraclass correlation coefficients (ICC; two-way mixed model, absolute agreement) were computed in SPSS v28.0 and interpreted as poor (<0.50), moderate (0.50–0.75), good (0.75–0.90), or excellent (>0.90). All ICC values were in the excellent range (0.91–0.97; all *p* < 0.001).

### 2.6. Statistical Analysis

Continuous variables were described as mean ± SD, median, IQR, and range. Normality was assessed with the Shapiro–Wilk test. MA–lingula distances were compared between paired bilateral non-retrotuberosity superficial sides using the paired *t*-test and the Wilcoxon signed-rank test. Independent group comparisons used Welch’s *t*-test and the Mann–Whitney U test. Associations between looping and close MA–lingula proximity were tested using Fisher’s exact test (threshold < 5 mm). Proportional side-agreement used McNemar’s test. Bilateral type distributions were compared with the chi-squared test. Age differences across types were assessed using the Kruskal–Wallis test. Correlations were computed using Pearson’s *r* and Spearman’s ρ. Because the two sides of a patient are not fully independent, all inferential comparisons of primary interest were performed within-subject (paired *t*-test, Wilcoxon signed-rank, and McNemar tests), whereas side-level values were used only for descriptive normative reporting, consistent with the prior maxillary-artery morphometric literature. All *p* < 0.05 were considered statistically significant. Analyses were performed in Python 3.11 (SciPy 1.11, StatsModels 0.14). Given the exploratory, hypothesis-generating nature of most comparisons, no global correction for multiple testing was applied; the pre-specified primary loop-proximity association and the sex dimorphism in MA–gonial angle distance retain significance under conservative Bonferroni adjustment, whereas the borderline Type 1 vs. Type 3 notch-position difference (*p* = 0.025) and the right–left MA–lingula laterality (*p* = 0.023) are reported as exploratory and would not survive strict correction.

## 3. Results

### 3.1. Sample Characteristics

The study included 164 patients (328 sides): 97 males (59.1%) and 67 females (40.9%). Mean age was 67.2 ± 13.0 years (median 68.5, IQR 59.0–76.0, range 25–91). Age did not differ significantly between sexes (males 66.7 ± 13.7 yr vs. females 67.9 ± 11.9 yr; *p* = 0.851). 

### 3.2. Variable 1: MA–Gonial Angle Distance

Valid measurements were obtained for 326/328 sides (two were excluded due to artefacts). The MA–GA distance was 34.53 ± 5.31 mm overall (median 34.25, IQR 30.30–38.27, range 22.6–48.5 mm; Shapiro–Wilk W = 0.982, *p* = 0.621). Right-side (34.79 ± 4.93 mm) and left-side (34.27 ± 5.66 mm) values did not differ significantly (Wilcoxon *p* = 0.186).

A significant sex dimorphism was identified: males, 36.74 ± 4.95 mm; females, 31.35 ± 4.04 mm (Mann–Whitney U, *p* < 0.0001). No correlation was found between MA–GA and age (Spearman r = −0.006, *p* = 0.942). MA–GA did not differ between superficial and deep-course sides (*p* = 0.287) or between sides with and without looping (*p* = 0.828), indicating that the sex effect reflects mandibular rather than vascular dimensions.

### 3.3. Variable 2: MA–Mandibular Notch Position (Superficial Sides)

The MA–MN directional position was recorded in 199/200 superficial sides. The mean was 2.57 ± 3.19 mm (median 2.70, IQR 0.78–4.50, range −8.60 to +11.40 mm). The MA was superior to the notch plane in 166/199 sides (83.4%), precisely at the notch in six (3.0%), and inferior in 27 (13.6%). The absolute magnitude was 3.37 ± 2.33 mm. No significant right–left (paired *t*-test *p* = 0.804) or sex (*p* = 0.737) differences were found. The absolute MA–MN magnitude was significantly greater in Type 1 than in Type 3 superficial sides (3.54 ± 2.38 vs. 2.68 ± 2.01 mm; Welch *p* = 0.025).

### 3.4. Variable 3: MA Looping in the ITF

Overall prevalence: Looping was present in 48 right sides (29.3%) and 45 left sides (27.4%; McNemar *p* = 0.664). At least one looping side was observed in 57 of 164 patients (34.8%), with bilateral looping in 36 (22.0%). Looping was not associated with sex (χ^2^ = 0.164, *p* = 0.686).

**Figure 2:**
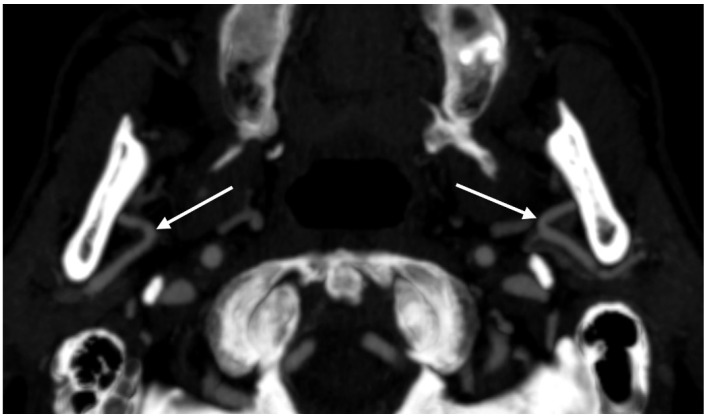
Axial CTA slice viewed inferiorly. Bilateral medial loops (arrows) of the maxillary arteries.

**Figure 3:**
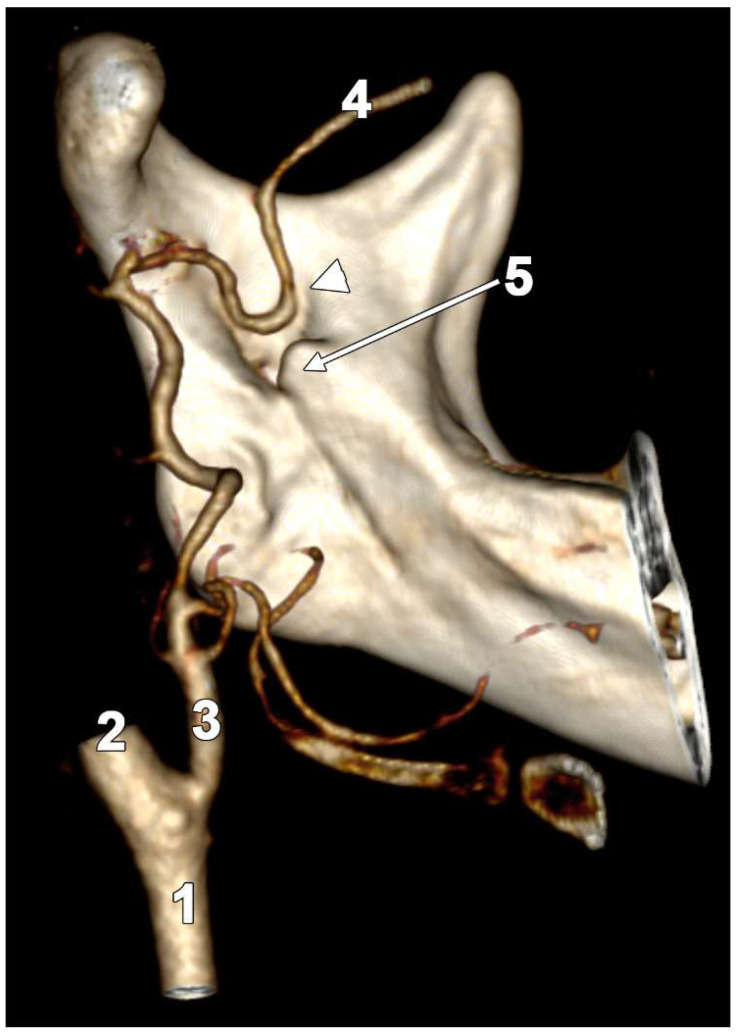
Three-dimensional volume rendering, left side, medial view of the mandibular ramus. An inferior loop (arrowhead) of the maxillary artery reaches 2.1 mm to the mandibular lingula. (1) Common carotid artery; (2) internal carotid artery; (3) external carotid artery; (4) maxillary artery; (5) mandibular lingula.

Topographic classification: Of 93 looped sides total, 32 sides from 19 cases carried a retrotuberosity label (22 superficial, 10 deep) and were excluded from lingula analyses. The remaining 61 non-retrotuberosity sides comprised interpterygoid triangle loops: medial loops (Figure 2) (n = 42), inferior loops (Figure 3) (n = 12), U-loops (n = 4), reversed U-loop (n = 1), coil (n = 1), and combined medial + inferior (n = 1). Non-retrotuberosity loop prevalence was significantly higher in Type 1 than Type 3 patients (33/80, 41.2% vs. 6/40, 15.0%; Fisher OR = 3.98, *p* = 0.004).

### 3.5. Variable 4: MA Course and Bilateral Topographic Types

The MA was superficial in 200/328 sides (61.0%) and deep in 128/328 sides (39.0%). No transpterygoid course was observed. Superficial courses were evenly distributed across sides, with 100 on each side; binomial *p* = 1.000. The prevalence of a superficial MA course did not differ significantly by sex: 61.9% in males and 59.7% in females; χ^2^ = 0.077, *p* = 0.781.

**Figure 4:**
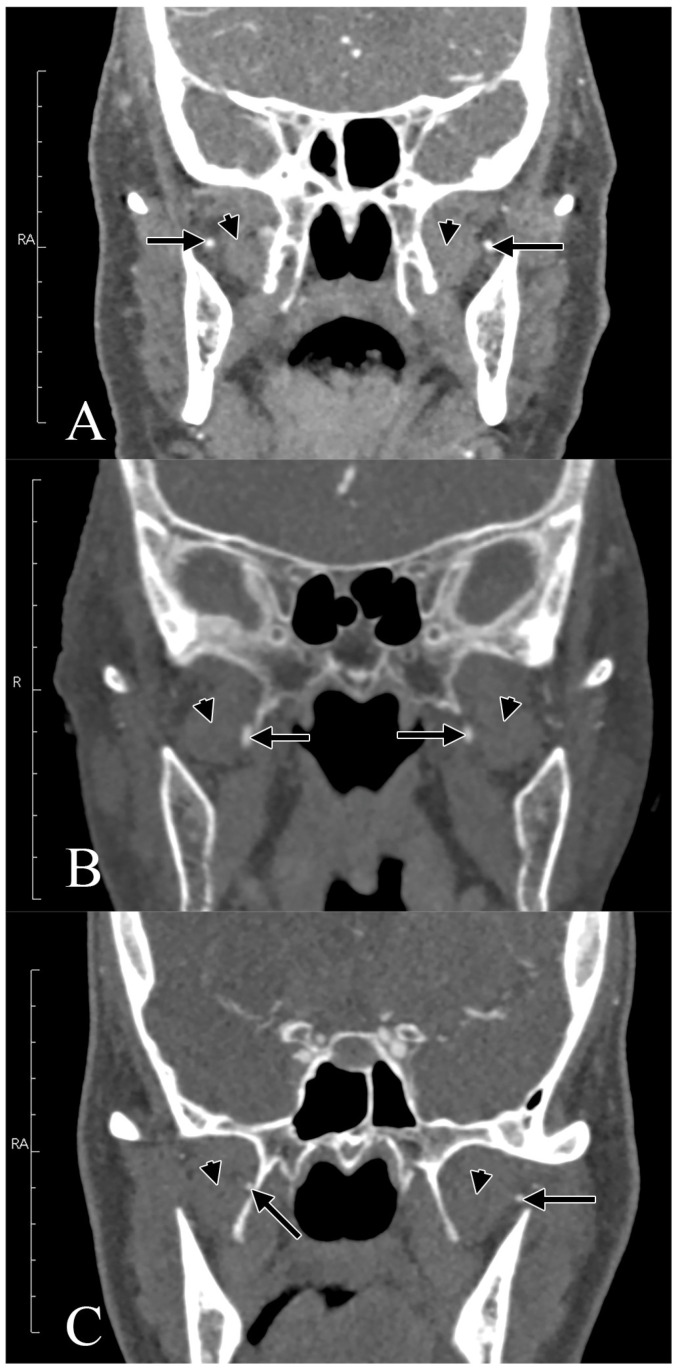
Bilateral combinations of maxillary arteries’ (arrows) courses, as referred to the lateral pterygoid muscles (arrowheads). Coronal MPR slices, viewed anteriorly. (**A**) Type 1—bilateral superficial (S) courses. (**B**) Type 2—bilateral deep (D) courses. (**C**) Type 3 courses—S + D.

Based on combinations of bilateral courses, three topographic types were identified (Figure 4): Type 1 (S + S) in 80 patients (48.8%), Type 2 (D + D) in 44 (26.8%), and Type 3 (S + D) in 40 (24.4%). Type 3 was perfectly balanced (20 right-S/left-D; 20 right-D/left-S). Bilateral type did not associate significantly with sex (χ^2^ = 4.38, *p* = 0.111) or age (Kruskal–Wallis *p* = 0.344) (Table 1).

### 3.6. Variable 5: MA–Lingula Distance (Non-Retrotuberosity Superficial Sides)

After exclusion of retrotuberosity, 178 superficial non-RT sides were eligible; valid MA–Ling measurements were available for 176 sides (R = 88, L = 88). Mean MA–Ling was 8.03 ± 2.54 mm (median 8.10, IQR 6.17–9.77, range 2.10–14.90 mm). Sex did not significantly influence MA–Ling (males 8.19 ± 2.69 mm vs. females 7.63 ± 2.42 mm; Welch *p* = 0.158) (Table 2).

Paired bilateral analysis (Type 1, non-retrotuberosity; n = 70 pairs): Right-side MA–Ling (7.70 ± 2.53 mm) was significantly shorter than the left-side (8.30 ± 2.74 mm; mean difference −0.60 ± 2.15 mm; paired t *p* = 0.023; Wilcoxon *p* = 0.028), indicating that the right MA consistently approaches the lingula more closely than the left in bilaterally superficial patients.

Clinical proximity thresholds: 24/176 sides (13.6%) had MA–Ling <5 mm; 3/176 (1.7%) had <3 mm. The minimum distance was 2.10 mm.

Association with looping: Loop-positive sides had significantly shorter MA–Ling than loop-negative sides (6.86 ± 2.83 mm vs. 8.64 ± 2.16 mm; Welch *p* < 0.0001; Mann–Whitney U *p* < 0.0001). Of the 24 sides with MA–Ling <5 mm, 19 (79.2%) were loop-positive. Fisher’s exact test confirmed a strong association: OR = 9.95, *p* < 0.000005. Looping was not associated with MA–GA (*p* = 0.828) or MA–MN position (*p* = 0.645), confirming that the loop–lingula relationship is topographically specific (Table 3).

### 3.7. Correlations Among Landmark Measurements

In the 176 non-retrotuberosity superficial sides with complete measurements, MA–Ling showed a weak but significant positive correlation with MA–GA (Pearson r = 0.219, *p* = 0.004; Spearman ρ = 0.221, *p* = 0.003) and with directional MA–MN position (Pearson r = 0.197, *p* = 0.009; Spearman ρ = 0.158, *p* = 0.036), indicating that arteries situated further from the gonial angle and superior to the notch also tend to be further from the lingula (Table 4).

## 4. Discussion

**Figure 5:**
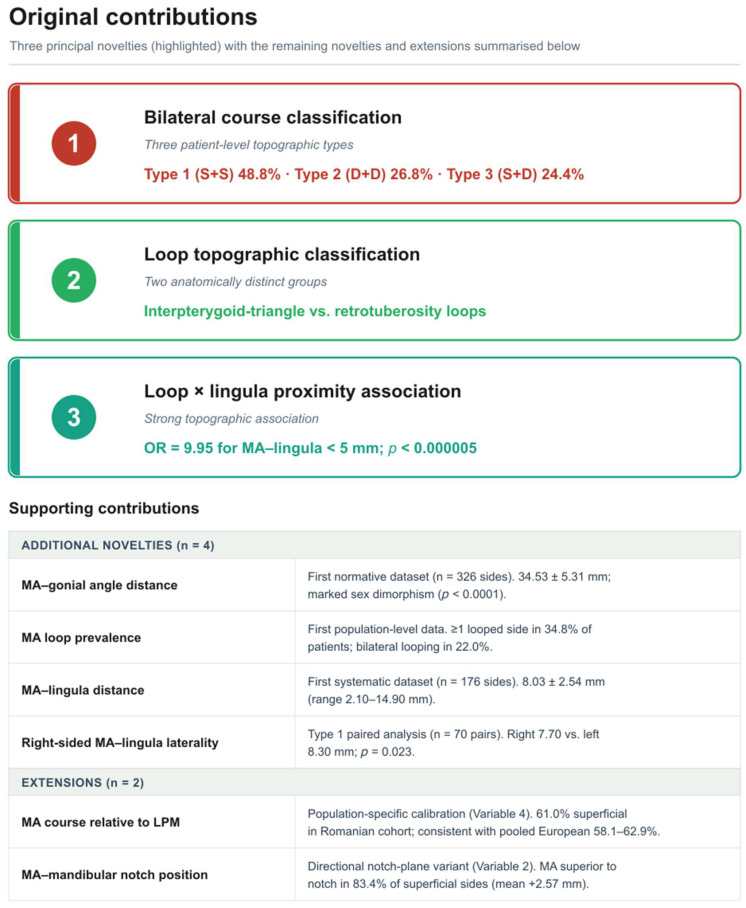
Original contributions of the present study, arranged as a top-to-bottom stack. The three principal novelties are highlighted as individual cards (top): (1) a three-type bilateral classification of the maxillary artery (MA) course relative to the lateral pterygoid muscle (LPM)—Type 1 (S + S) 48.8%, Type 2 (D + D) 26.8%, Type 3 (S + D) 24.4% (red); (2) a topographic classification of MA looping into interpterygoid triangle and retrotuberosity groups (green); and (3) a strong topographic association between non-retrotuberosity looping and dangerous MA–lingula proximity, with a nearly tenfold increase in the odds ratio (OR = 9.95 for MA–lingula < 5 mm; *p* < 0.000005) (teal). Each card reports the corresponding key quantitative result. The remaining contributions are summarised in the panel below: four additional novelties—the first normative MA–gonial angle dataset (34.53 ± 5.31 mm; n = 326 sides; sex dimorphism *p* < 0.0001), the first population-level MA loop prevalence (≥1 looped side in 34.8%; bilateral 22.0%), the first systematic MA–lingula dataset (8.03 ± 2.54 mm; n = 176 sides), and a previously unreported right-sided MA–lingula laterality in Type 1 patients (right 7.70 vs. left 8.30 mm; *p* = 0.023)—together with two extensions of established variables: population-specific calibration of the MA course relative to the LPM (Variable 4; 61.0% superficial, consistent with pooled European data) and a directional notch-plane variant of the MA–mandibular notch position (Variable 2; MA superior to the notch in 83.4% of superficial sides). S = superficial/lateral to the LPM; D = deep/medial to the LPM; MA = maxillary artery; LPM = lateral pterygoid muscle; OR = odds ratio.

The present study advances the existing literature on the maxillary artery (MA) in nine distinct ways, summarised schematically in Figure 5. Seven of these are novel contributions; the remaining two extend previously established variables to a Romanian CTA cohort. The novelties are: (i) a three-type patient-level classification of the bilateral MA–LPM course—Type 1 (S + S), Type 2 (D + D), and Type 3 (S + D), accounting for 48.8%, 26.8%, and 24.4% of patients, respectively; (ii) a topographic separation of MA loops into interpterygoid triangle and retrotuberosity groups, treated here as anatomically and analytically distinct entities; (iii) the first normative dataset of the MA–gonial angle distance (34.53 ± 5.31 mm; n = 326 sides), with a marked sex dimorphism (*p* < 0.0001) that proved unrelated to MA course or loop status; (iv) the first population-level prevalence data for MA looping in the infratemporal fossa, with at least one looped side in 34.8% of patients and bilateral looping in 22.0%; (v) the first systematic measurement of the MA–lingula distance (8.03 ± 2.54 mm; n = 176 non-retrotuberosity superficial sides); (vi) a previously unreported right-sided laterality of the MA–lingula distance in Type 1 patients (right 7.70 vs. left 8.30 mm; *p* = 0.023); and (vii) a strong topographic association whereby non-retrotuberosity looping confers a nearly tenfold increase in the odds of dangerous (<5 mm) MA–lingula proximity (OR = 9.95; *p* < 0.005). The two extensions are the population-specific calibration of the MA course relative to the LPM (Variable 4) and the directional notch-plane variant of the MA–mandibular notch position (Variable 2), both of which place our cohort within the existing meta-analytic literature. Taken together, these findings define a coherent topographic framework for the infratemporal MA on CTA; the embryological substrate underlying the looping and fenestration patterns documented here is examined in the following section.

### 4.1. Embryological Development of the Maxillary Artery

The adult MA results from the complex remodelling of transient embryonic vessels, including the pharyngeal arch arteries, the stapedial system, and primitive cranial arterial plexuses. Understanding these developmental pathways directly contextualises the adult morphological variants documented in the present and prior studies. The extracranial arterial system, including the external carotid artery and its branches, is fundamentally derived from the pharyngeal arch arteries, predominantly the second (hyoid) arch [15]. Early in embryogenesis, the primitive internal carotid artery extends cranially and gives rise to the primitive maxillary artery, which transiently supplies the premandibular and optic regions before largely regressing [16]. Subsequent foetal dissection studies have shown that the vessel historically termed the “primitive maxillary artery” is more accurately interpreted as the cavernous inferolateral trunk of the ICA rather than a true precursor of the extracranial adult maxillary artery, since no separate persistent primitive maxillary artery was identified in dissectible foetal specimens [17,18].

The definitive extracranial maxillary artery originates primarily from the maxillomandibular division of the stapedial artery, a transient vessel arising from the hyoid (second pharyngeal) arch and traversing the developing stapes, in combination with annexation by the ventral pharyngeal artery [19,20]. The stapedial artery’s maxillomandibular branch carries the precursors of the infraorbital and inferior alveolar arteries. When the ventral pharyngeal artery subsumes this branch, it establishes the external carotid trunk and the proximal maxillary artery, whilst the stapedial trunk itself largely regresses, leaving permanent remnants such as the caroticotympanic and superior tympanic arteries [19,20]. The middle meningeal artery is itself a derivative of the stapedial maxillomandibular and supraorbital branches, a lineage that explains the well-documented variant of MMA origin from the ophthalmic artery when the stapedial–ophthalmic anastomosis persists [19,20,21].

Directly relevant to the MA–LPM topographic variants described in this and prior studies, the developing maxillary artery is established embryologically as a vascular ring around the mandibular nerve, comprising superficial and deep components [22]. In the normal developmental sequence, one component regresses, yielding either a superficial or a deep adult course relative to the LPM. Persistence of both ring halves produces a duplicated or divided-and-reunited arterial configuration [22,23,24], whilst intermediate degrees of ring persistence likely underlie the looping configurations documented at the population level in the present study. Foetal anatomical studies confirm that this ring architecture is already established in the perinatal period and that its variability accounts for the full adult MA morphological spectrum [25].

The present study introduces a three-type bilateral classification of the MA–LPM relationship—Type 1 (S + S), Type 2 (D + D), and Type 3 (S + D)—which, to our knowledge, has not been formalised in prior studies. Although bilateral asymmetry in the MA course has been noted incidentally [6,7,26], the existing literature, including two large meta-analyses encompassing nearly 6000 arteries [4,5], has consistently treated the MA–LPM relationship as a per-side or pooled-side variable, without characterising patients by their bilateral patterns. Our data show that Type 1 predominates (48.8%), followed by Type 2 (26.8%) and Type 3 (24.4%). The perfect balance of Type 3, 20 right-S/left-D and 20 right-D/left-S cases, argues that left–right asymmetry in the MA course is a stochastic developmental event rather than a lateralised trait. The practical implication is that a surgeon planning a unilateral procedure for a Type 3 patient cannot infer the operative-side MA position from contralateral imaging, underscoring the value of side-specific preoperative CTA in all cases, as has been advocated for orthognathic and condylar procedures [27,28].

### 4.2. MA–Gonial Angle Distance: First Normative Dataset

The MA–GA distance of 34.53 ± 5.31 mm (n = 326 sides) constitutes the first normative dataset for this relationship. Existing studies have used the mandibular notch [8,9,10], condylar apex and articular eminence [9,29], and pterygoid fovea [10] as bony references, but none have used the gonial angle as a systematic measurement variable. The sole precedent is a descriptive CTA case report noting that the terminal bifurcation of a maxillofacial trunk occurred 1.47 cm deep to the gonial angle [14], confirming the landmark’s visibility on CTA but providing no population-level reference data. The marked sex dimorphism, males exceeding females by a mean of 5.4 mm (36.74 vs. 31.35 mm; *p* < 0.0001), is consistent with well-documented mandibular sexual dimorphism and indicates that MA–GA distance is primarily a function of jaw dimensions rather than intrinsic vascular topography, given the absence of correlation with age (r = −0.006) and the absence of any difference between superficial and deep-course sides or loop status. The gonial angle’s principal advantage over previously used landmarks is its consistent identifiability on panoramic radiography, the most widely used preoperative imaging modality in dentistry and oral surgery, as well as on 3D CT, making it practically accessible across clinical settings with varying imaging resources.

### 4.3. Looping of the MA in the ITF: First Population-Level Prevalence and Novel Topographic Classification

The prevalence of MA looping in the ITF, at least one looping side in 34.8% of patients and bilateral looping in 22.0%, is the most epidemiologically novel finding of this study. Before this work, MA looping had been documented exclusively in isolated CTA case reports: Rusu et al. (2019) described gamma and U-loops of the mandibular and pterygoid segments approaching the lingula in a patient investigated for subarachnoid haemorrhage [12]; Rusu et al. (2024) reported a sagittal loop applied to the lingula, 0.5 cm above the mandibular foramen [11]; and Rusu et al. (2026) documented tortuous MA configurations associated with compensatory vascular patterning [13]. None of these reports permitted population-level inference. Our data establish that looping is not a rare curiosity but a common variant, present in more than one in three European patients on CTA.

The two-group topographic classification introduced here, interpterygoid triangle loops and retrotuberosity loops, is anatomically and surgically meaningful and distinguishes configurations that have been conflated in case reports. Interpterygoid triangle loops, which dominated (medial loops accounting for 42 of 61 non-retrotuberosity sides), direct the MA into the space enclosed by the medial and lateral pterygoid muscles, in close proximity to the IAN, lingual, and buccal nerves, the same structures at risk during IAN blocks, sagittal split ramus osteotomy, and condylar surgery [26,30,31]. Retrotuberosity loops, by contrast, direct the vessel posteriorly behind the maxillary tuberosity, into the operative field for pterygomaxillary disjunction, Le Fort I osteotomy, and tuberosity-level haemostasis [28,32]. Consequently, the topographic loop type determines which surgical procedure is at greatest specific risk, and the two groups therefore warrant separate clinical attention. The clinical weight of the MA course for needle-based access to the pterygopalatine and infratemporal fossae was recently reinforced by a three-dimensional simulation study in children, in which the maxillary artery frequently intersected infrazygomatic needle trajectories to the pterygopalatine fossa and rendered the anterior infrazygomatic maxillary nerve block unsafe in the majority of simulated attempts, whereas the more cranial suprazygomatic route was almost always safe [33]; accordingly, detection of an interpterygoid loop on preoperative imaging should prompt reconsideration of the needle trajectory and, where available, image guidance for maxillary nerve block and related pterygopalatine fossa procedures. The anatomical basis for these configurations most likely reflects the persistence of embryological vascular rings in the developing infratemporal fossa [23,24,34], as evidenced by cadaveric reports of divided and reunited maxillary arteries forming complete loops within the infratemporal fossa, in which partial or complete ring retention produces the morphological equivalent of the looping patterns documented here at the population level.

The finding that non-retrotuberosity loops are significantly more prevalent in Type 1 than in Type 3 patients (41.2% vs. 15.0%; OR = 3.98, *p* = 0.004) is entirely novel. It implies that bilaterally superficial patients, already the most common bilateral type and those most directly exposed to IAN block and ramus surgery hazards, also harbour the highest interpterygoid loop burden, compounding their vascular risk. This interaction between bilateral course type and loop prevalence has not previously been investigated in any study.

### 4.4. MA–Lingula Distance: First Systematic Measurement, Right-Side Laterality, and Loop Association

The MA–lingula distance of 8.03 ± 2.54 mm (range 2.10–14.90 mm) on 176 non-retrotuberosity superficial sides constitutes the first population-level dataset for this clinically critical proximity. Prior lingula-centred vascular data were limited to two single-case CTA reports: Rusu et al. (2024) described a loop “applied on the lingula” at 0.5 cm above the mandibular foramen, and Rusu et al. (2026) documented an aberrant buccal artery entering the mandibular ramus precisely at the lingula [11,13]. Balcioglu et al. (2010) measured the lingula’s position relative to bony landmarks of the ramus but did not measure the MA–lingula distance itself [9]. Hwang et al. (2014), although highly relevant as a 3D CT comparator for MA–notch and ramus relationships, likewise did not measure the MA–lingula distance [8]. These precedents position our dataset as the first normative reference for direct MA–lingula measurement, relevant to IAN block safety and sagittal split osteotomy planning [26,31].

Two findings for this variable merit particular emphasis. First, the paired bilateral analysis of Type 1 patients (n = 70 pairs) showed that the right MA is significantly closer to the lingula than the left (7.70 ± 2.53 vs. 8.30 ± 2.74 mm; mean difference −0.60 mm; *p* = 0.023). This right-sided laterality is a previously unreported asymmetry. Given that right-sided IAN blocks are the most common dental injection among right-dominant clinicians, this finding has measurable implications for anaesthetic safety. However, its anatomical basis, whether related to mandibular morphological asymmetry, pterygoid venous plexus laterality, or developmental factors, requires further investigation.

Second, the association between non-retrotuberosity looping and close MA–lingula proximity is of exceptional clinical magnitude. Among loop-positive sides, 31.1% had MA–Ling <5 mm, compared with only 4.3% of loop-negative sides (Fisher OR = 9.95, *p* < 0.000005). The nearly tenfold increase in the odds of dangerous proximity means that the detection of an interpterygoid loop on preoperative CTA should prompt reconsideration of the standard IAN block technique and heightened surgical caution during lingual-surface ramus procedures, irrespective of the absolute measured distance in that patient. The minimum recorded distance of 2.10 mm represents an extremely close MA–lingula relationship in which conventional IAN block technique may carry a substantial risk of arterial puncture or intra-arterial injection, with attendant risk of local anaesthetic systemic toxicity and inadvertent CNS injection [30]. The 1.7% of sides with MA–Ling <3 mm represent patients for whom alternative anaesthetic techniques, such as ultrasound-guided approaches [30], should be seriously considered.

### 4.5. MA–Mandibular Notch Position: Prevalence and Surgical Safety

The demonstration that the MA lies superior to the mandibular notch plane in 83.4% of superficial-course sides (mean +2.57 mm) extends prior landmark studies by applying a signed directional notch-plane measurement in a European CTA cohort. Published measurements are not directly interchangeable because they used different anatomical endpoints and measurement definitions: Balcioglu et al. (2010) reported a mean MA-to-notch distance of 2.94 ± 0.52 mm in Turkish cadavers, whereas Hwang et al. (2014) provided the closest imaging comparator by reconstructing the MA, LPM and mandibular ramus in 100 Korean patients [8,9]. In Hwang’s lateral-course group, the shortest MA–mandibular notch distance was 3.6 ± 1.0 mm. In contrast, the medial-course group showed a substantially larger distance of 16.3 ± 3.7 mm, confirming that the lateral/superficial MA is the clinically relevant configuration for ramus-related vascular risk. Their contact-point analysis also showed that the MA–mandible relationship varies in both vertical and anteroposterior directions relative to the mandibular notch. However, this was not identical to the directional notch-plane variable used here. Our wider range (−8.60 to +11.40 mm) and the 3.0% of superficial sides at the notch level therefore extend rather than duplicate the known distribution and support case-by-case preoperative imaging, as advocated for orthognathic and condylar procedures [27,35]. The finding that absolute MA–MN magnitude is significantly greater in Type 1 than Type 3 superficial sides (3.54 vs. 2.68 mm; *p* = 0.025) suggests that bilaterally superficial patients have a modestly higher positional displacement of the MA relative to the notch, a further argument for bilateral topographic typing in surgical planning. Complications of unrecognised MA proximity to the notch, including life-threatening haemorrhage requiring ECA ligation, have been reported [36].

### 4.6. MA Course Relative to the LPM: Comparison with the Literature

The 61.0% superficial prevalence in this Romanian population is consistent with pooled White/European data of 58.1–62.9% from Wang et al. (2018) and Piagkou et al. (2025) and contrasts with the higher values reported in Asian cohorts, including the 82.0% lateral-course prevalence in Hwang et al.’s Korean 3D CT series [5,8] and the pooled Asian rate of approximately 90.9% [3]. Historical European cadaveric studies show a wide range: Lurje et al (1946) found 67.5% in 200 preparations; Pretterklieber et al. (1991) reported 55.4% in 102 individuals; Hussain et al. (2008) found 68.2% in Caucasians; and Dennison et al. (2009) documented population-specific differences in a New Zealand cohort [26,37,38,39]. The absence of an intramuscular (through) course is consistent with the 1–3% prevalence, which would require a substantially larger sample size to detect reliably [6]. The 25.0% bilateral asymmetry rate (Type 3) aligns with the 21.1% documented by Gulses et al. (2012) in 209 CTA patients and the approximately 26% in Albu et al. (2025), supporting our three-type classification against published prevalence data [6,7]. The lack of a sex association with the MA course is consistent with most cadaveric studies [10,39], although Albu et al. (2025) found a higher rate of deep course in females when specific LPM morphological types, themselves sex-associated, were considered; this interaction could not be assessed without LPM morphological data in the present dataset [6].

### 4.7. Limitations

The present study has several limitations. First, the retrospective design and single-centre data source limit generalisability; prospective multicentre replication across European populations is needed. Second, LPM morphological typing (one to three heads) was not performed, precluding analysis of the MA–LPM–loop interaction described previously [6,11]. Third, the CTA datasets were not acquired specifically for anatomical research; resolution limitations may have affected the detection of minor looping configurations or small-calibre variant branches. Fourth, interobserver agreement was assessed in 20% of cases by a single second observer; formal intraobserver assessment was not performed. Fifth, although the key comparisons were paired within-subject, side-level descriptive statistics do not formally model within-patient clustering, and a mixed-effects framework would be preferable in a larger prospective cohort. Sixth, the exploratory comparisons were not corrected for multiple testing and require independent confirmation. Despite these limitations, this study represents the largest CTA-based bilateral analysis of MA topography in a European population. It provides the first population-level data on the prevalence of looping, MA–lingula distance, and MA-gonial angle distance.

## 5. Conclusions

In this CTA series, the MA most frequently followed a superficial course relative to the lateral pterygoid muscle. Classifying patients by bilateral arterial pattern identified three configurations: bilateral superficial, bilateral deep, and asymmetric. This approach adds clinically useful information beyond isolated side-based reporting.

The MA–gonial angle distance showed a clear sex-related difference, with larger values in males, while MA looping was common enough to merit routine attention during image review. Separating loops into interpterygoid triangle and retrotuberosity patterns is anatomically relevant because these configurations relate to different operative corridors and different potential sites of vascular injury.

The closest relationship to the mandibular lingula was observed in the non-retrotuberosity superficial arteries, especially when looping was present. These loops were strongly associated with MA–lingula distances below 5 mm, indicating a higher-risk arrangement for inferior alveolar nerve block, sagittal split ramus osteotomy, subcondylar surgery, and other procedures along the medial mandibular ramus. Preoperative CTA recognition of the arterial course, loop pattern, and lingular proximity may help identify patients requiring modified anaesthetic or surgical planning.

## Figures and Tables

**Table 1:** Demographics and bilateral course combinations. MA = maxillary artery; S = superficial; D = deep; M = male; F = female.

Variable	Result
Cases/sides	164/328
Sex	97 males (59.1%); 67 females (40.9%)
Age (years)	Mean 67.2 ± 13.0; median 68.5 (IQR 59.0–76.0); range 25–91
Superficial MA course	200/328 sides (61.0%)
Deep MA course	128/328 sides (39.0%)
Transpterygoid course	0/328 sides
Type 1—bilateral superficial (S + S)	80/164 cases (48.8%); M = 51, F = 29; mean age 68.4 ± 12.4 yr
Type 2—bilateral deep (D + D)	44/164 cases (26.8%); M = 28, F = 16; mean age 66.3 ± 13.7 yr
Type 3—asymmetric (S + D or D + S)	40/164 cases (24.4%); M = 18, F = 22; mean age 65.7 ± 13.3 yr
Type 3 sub-distribution	Right-S/Left-D: 20; Right-D/Left-S: 20
Bilateral type × sex (χ^2^)	χ^2^ = 4.38, *p* = 0.111
Bilateral type × age (Kruskal–Wallis)	*p* = 0.344

**Table 2:** Landmark morphometry in superficial MA variants. MA–MN directional position: positive = MA is superior to the notch; negative = MA is inferior to the notch. MA–lingula restricted to the non-retrotuberosity superficial sides. Overall right/left rows use all valid non-RT superficial sides (n = 88 per side). Type 1 paired rows report the paired bilateral analysis (n = 70 pairs).

Measurement	n	Mean ± SD	Median (IQR)/Range
MA–gonial angle distance (all sides, bilateral)	326	34.53 ± 5.31 mm	34.25 (30.30–38.27)/22.6–48.5 mm
Males	194	36.74 ± 4.95 mm	*p* < 0.0001 vs. females (MW)
Females	132	31.35 ± 4.04 mm	
MA–mandibular notch position (directional; superficial sides)	199	2.57 ± 3.19 mm	2.70 (0.78–4.50)/−8.60 to +11.40 mm
MA–mandibular notch-absolute magnitude	199	3.37 ± 2.33 mm	3.00 (1.40–5.10)/0.00–11.40 mm
MA–lingula distance (non-RT superficial sides; valid measurements)	176	8.03 ± 2.54 mm	8.10 (6.17–9.77)/2.10–14.90 mm
Overall right side (valid non-RT superficial sides)	88	7.74 ± 2.35 mm	
Overall left side (valid non-RT superficial sides)	88	8.32 ± 2.71 mm	
Type 1 paired right side	70 pairs	7.70 ± 2.53 mm	
Type 1 paired left side	70 pairs	8.30 ± 2.74 mm	paired t *p* = 0.023; Wilcoxon *p* = 0.028

**Table 3:** Loop analysis and MA–lingula proximity. NRT *=* non-retrotuberosity. OR = odds ratio (Fisher’s exact test). ns = not significant.

Variable	Result
Total looped sides (any loop)	93 sides (48 R + 45 L); ≥1 side in 57/164 patients (34.8%); bilateral in 36 (22.0%)
Retrotuberosity-labelled sides excluded	32 sides from 19 cases (22 superficial, 10 deep)
Eligible superficial non-RT sides (MA–Ling analysis)	178 sides (176 with valid measurements)
Non-RT loop-positive sides	61 sides (R = 31, L = 30)
Non-RT loop-positive cases (≥1 eligible side)	39/108 cases (36.1%)
Non-RT loop morphologies	Medial loop 42; inferior loop 12; U-loop 4; reversed U 1; coil 1; medial + inferior 1
NRT loop prevalence—Type 1 vs. Type 3	Type 1: 33/80 (41.2%) vs. Type 3: 6/40 (15.0%); Fisher OR = 3.98, *p* = 0.004
MA–lingula: loop-positive vs. loop-negative sides	6.86 ± 2.83 mm vs. 8.64 ± 2.16 mm; Welch *p* < 0.0001; MW *p* < 0.0001
Sides with proximity (<5 mm) to lingula	24/176 (13.6%); of these, 19/24 were loop-positive
Fisher’s exact test (NRT loop × <5 mm proximity)	OR = 9.95; *p* < 0.000005
Sides with very close proximity (<3 mm) to the lingula	3/176 (1.7%); minimum recorded distance 2.10 mm
Loop-positive vs. loop-negative MA–GA distance	34.92 ± 5.52 mm vs. 35.10 ± 5.42 mm; *p* = 0.828 (ns)
Loop-positive vs. loop-negative MA–MN position	2.40 ± 3.48 mm vs. 2.64 ± 3.07 mm; *p* = 0.645 (ns)

**Table 4:** Correlations among landmark measurements (n = 176 non-RT superficial sides). All correlations were computed on the non-retrotuberosity superficial sides with complete data for all three variables.

Pair (Non-RT Superficial Sides, n = 176)	Pearson r	*p*	Spearman ρ	*p*
MA–lingula vs. MA–gonial angle	0.219	0.004	0.221	0.003
MA–lingula vs. MA–MN directional position	0.197	0.009	0.158	0.036

## Data Availability

The datasets generated and analysed during the current study are available from the corresponding author upon reasonable request.
